# Seasonal variation in denitrification and dissimilatory nitrate reduction to ammonia process rates and corresponding key functional genes along an estuarine nitrate gradient

**DOI:** 10.3389/fmicb.2015.00542

**Published:** 2015-06-02

**Authors:** Cindy J. Smith, Liang F. Dong, John Wilson, Andrew Stott, A. Mark Osborn, David B. Nedwell

**Affiliations:** ^1^Department of Biological Sciences, University of Essex, Colchester, UK; ^2^Department of Animal and Plant Sciences, The University of Sheffield, Sheffield, UK; ^3^NERC Life Sciences Mass Spectrometry Facility, Centre for Ecology and Hydrology, Lancaster Environment Centre, Lancaster, UK

**Keywords:** denitrification/DNRA, *narG*, *napA*, *nirS*, *nrfA*, (RT)-Q-PCR

## Abstract

This research investigated spatial-temporal variation in benthic bacterial community structure, rates of denitrification and dissimilatory nitrate reduction to ammonium (DNRA) processes and abundances of corresponding genes and transcripts at three sites—the estuary-head, mid-estuary and the estuary mouth (EM) along the nitrate gradient of the Colne estuary over an annual cycle. Denitrification rates declined down the estuary, while DNRA rates were higher at the estuary head and middle than the EM. In four out of the six 2-monthly time-points, rates of DNRA were greater than denitrification at each site. Abundance of gene markers for nitrate-reduction (nitrate reductase *narG* and *napA*), denitrification (nitrite reductase *nirS*) and DNRA (DNRA nitrite reductase *nrfA*) declined along the estuary with significant relationships between denitrification and *nirS* abundance, and DNRA and *nrfA* abundance. Spatially, rates of denitrification, DNRA and corresponding functional gene abundances decreased along the estuary. However, temporal correlations between rate processes and functional gene and transcript abundances were not observed.

## Introduction

Estuarine sediments are natural environmental gradients of nutrients and salinity, and significant sites of microbial diversity and activity. Bacterial diversity within these sediments is often higher than in other environments (Lozupone and Knight, 2007) and the bacteria present drive essential nutrient cycles with direct consequences for ecosystem function. Previously, we showed that the largest nutrient loads to all mainland UK estuaries were attributable to nitrate: at least an order of magnitude greater than ammonium (Nedwell et al., 2002; Earl et al., 2014). Benthic microorganisms mediate the nitrate load entering coastal waters via denitrification, dissimilatory nitrate reduction to ammonia (DNRA) and anaerobic ammonia oxidation (anammox) processes.

Heterotrophic denitrification by facultatively anaerobic bacteria using nitrate to respire organic matter, produces N_2_ and to a lesser extent the greenhouse gas N_2_O (Seitzinger, 1988; Nedwell et al., 1999; Dong et al., 2002), removing up to 50% of the nitrate load from estuaries (Nedwell et al., 1999). Anammox, the anaerobic autotrophic oxidization of NH_4_^+^, uses NO_2_^–^ as an electron acceptor yielding N_2_ (Strous et al., 1999; Kuenen, 2008). Reported values of N_2_ production from anammox in estuarine sediments range from 0 to 30% (Dong et al., 2009; Nicholls and Trimmer, 2009; Brin et al., 2014). Denitrification and anammox are significant pathways that remove nitrate as gaseous products from ecosystems, thus reducing the risk of eutrophication. In contrast, DNRA is an alternative pathway that reduces nitrate and nitrite to ammonium. It is a significant, but often overlooked processes in coastal benthic sediments accounting for up to 30% of nitrate reduction activity (Giblin et al., 2013). DNRA may contribute to eutrophication by retaining biologically available nitrogen within the system as NH_4_^+^. Thus the balance between the two predominant nitrate reduction pathways of denitrification and DNRA in benthic estuarine sediments influence the nutrient load entering costal waters.

Both denitrification and DNRA compete for NO_3_^–^ and NO_2_^–^ as an electron acceptor. Nitrogen transformations by denitrification and DNRA in estuarine sediments are influenced by interactions between a number of factors, primarily NO_3_^–^ and organic carbon concentrations, temperature and pH. Previous studies in estuarine sediments have indicated that denitrification may be favored when nitrate concentrations are high while DNRA tends to outcompete denitrification where there is high availability of organic electron donor and low nitrate (King and Nedwell, 1985; Dong et al., 2011).

Denitrification and DNRA are catalyzed by a series of nitrate and nitrite reductase enzymes encoded by genes whose abundance can be used as proxies in determining potential for nitrate reduction within environments (Philippot and Hallin, 2005). *narG* and *napA* genes encode subunits of two distinct nitrate reductases (NAR and NAP) that mediate reduction of nitrate to nitrite. Nitrite can enter the denitrification pathway in processes mediated by nitrite reductase enzymes encoded by *nirS* or *nirK* genes (see Zumft, 1997). Alternatively, in DNRA nitrite is reduced to NH_4_^+^ by the NrfA enzyme encoded by *nrfA* (see Simon, 2002). Consequently, molecular analyses of *nirS/nirK* and *nrfA* genes can be used to investigate the genetic potential in an environment for either denitrification or DNRA.

Previously, we demonstrated a decline in rates of denitrification and DNRA, and a corresponding decline in abundance of genetic markers for these processes, down the nitrate gradient of the Colne estuary (Dong et al., 2009) at a single time point (February 2005). Furthermore, previous research revealed seasonal variability in rates of denitrification (Dong et al., 2000); and spatial variability in rates of nitrate exchange across the sediment/water interface, with highest rates in the upper Colne estuary and lowest at the mouth (Thornton et al., 2007). These studies did not however, simultaneously study the fate of nitrate via benthic denitrification or DNRA nor the nitrate and nitrite reducing communities driving these processes over an annual period to determine seasonal effects. Therefore the aim of this study was to determine rates of nitrate reduction processes linked to corresponding functional gene and transcript abundances along the Colne estuary at 2 monthly intervals over a 12-month period. Based on previous studies we propose the following hypotheses: first, the trend of decreasing rates of denitrification and DNRA along the estuary gradient is seasonally stable. Second, the relative importance of denitrification and DNRA will vary seasonally, with DNRA higher in the summer. Third, that the abundance of key functional genes and transcripts will correlate spatially and seasonally with corresponding rate processes along the estuary.

## Materials and Methods

### Site Description, Field Sampling and Nutrient Analysis

Three sites along the Colne estuary, U.K. the estuary head (EH) at the Hythe, mid-estuary (ME) at Alresford Creek and the estuary mouth (EM) at Brightlingsea were sampled at 2-monthly intervals from April 2005 to February 2006. The EH is characterized by fine silt sediments (87–98% silt:clay < 65 μM) and salinity range between 2 and 17 ppt; ME sediments are fine silt (80–95% silt:clay < 65 μM), with salinity range between 20 and 32 ppt while EM sediments are clay with a thin layer of fine mud sand (silt:clay < 65 μM) and salinity ranging from 28 to 32 ppt. At each site replicate small cores of sediment (10 cm length in core tubes, 3.4 cm internal diameter by 22 cm length) were taken to measure process rates (*n* = 5 per each process). Triplicate sediment samples were also taken from the top 1 cm of sediment for molecular analysis, and returned on ice to the laboratory within 1 h of sampling, prior to storage at –70°C of aliquots (0.5 g wet weight) of sediment. Water samples were also collected at high tide at the three sites, then samples (10 ml) were filtered through glass fiber filter papers (GF/F, Whatman, UK) and frozen at –20°C prior to subsequent colorimetric nutrient analyses (Strickland and Parsons, 1972) using a segmented flow autoanalyzer (Skalar Analytical B.V., Breda, Netherlands). Limits of detection for nitrate and nitrite were 0.002 μM, ammonium 0.003 μM. Analytical accuracy for nutrient analysis was maintained by membership of a quality assurance scheme (www.quasimeme.org).

### Measurement of Rates of Denitrification and DNRA

Denitrification rates to both N_2_ and N_2_O (Dong et al., 2006) and DNRA rates (Dong et al., 2009) were determined simultaneously on intact sediment cores. Briefly, denitrification and DNRA rates were measured by ^15^N-labeled nitrate addition to sediment cores. Five cores of sediment (∼10 cm deep) were collected in Perspex tubes (3.4 cm internal diameter, 22 cm length) from each site. On return to the laboratory, the cores were put in an incubation tank at *in situ* water temperature and submerged in site water that was vigorously bubbled with air overnight to re-equilibrate. Next day, the rates of denitrification and DNRA were measured by ^15^N labeled nitrate addition to sediment cores. After a 3-h incubation of the cores, the sediment core and the overlying water were mixed to form a slurry. Slurry samples (12.5 ml) were removed for the quantification of denitrification gaseous products (N_2_ and N_2_O). Subsamples (10 ml) of the slurried sediment cores were taken for the subsequent recovery of ^15^NH_4_^+^ to determine rates of DNRA. Ammonium in the slurry samples was extracted by steam distillation and ammonium gas was trapped in acid solution. Ammonium in the acid solution was then absorbed onto zeolite, which was then combusted and reduced to N_2_. Isotope ratios of N_2_ in samples were measured by isotope ratio mass spectrometry using the ^14:15^N_2_ ratio in air as a standard. DNRA rates were calculated using the isotope ratio of N_2_. The DNRA calculation in the previous (Dong et al., 2009, 2011) and present work used the ratios of ^14:15^NO_3_^–^ in the water column, thus showing only the rate of DNRA supported by nitrate from the water column.

### Nucleic Acid Extraction

DNA and RNA were co-extracted from 0.5 g sediment, using Lysing Matrix B tubes (Bio-101) as described previously in Smith et al. (2007). Briefly, to each 0.5 g sediment sample, 0.5 ml of 240 mM sodium phosphate buffer (pH 8) and 0.5 ml of phenol:chloroform:isoamyl alcohol (25:24:1) (pH 4) were added. Samples were lysed by bead beating for 30 s at 2,000 rpm, and centrifuged for 10 mins at 17,563 × g. The aqueous phase was added to 0.5 ml chloroform:isoamyl (24:1), mixed and centrifuged for a further 10 min at 17,563 × g. The aqueous phase was removed for DNA and RNA precipitation with 2.5 volumes of ice-cold ethanol and 1/10 volume of 3 M sodium acetate (pH 5.2). Total nucleic acids were pelleted by centrifugation and washed twice in ice cold 70% ethanol, air dried and suspended in 100 μl of DEPC water. Total RNA was prepared by diluting a 25 μl aliquot of total nucleic acids with an equal volume of DEPC-treated sterile water, followed by digestion using TURBO DNA-free (Ambion, Austin, TX, USA) in accordance with the manufacturer’s protocol.

### 16S Ribosomal RNA Gene T-RFLP Analysis, Clone library Construction and Q-PCR Analysis

For Terminal Restriction Fragment Length Polymorphism (T-RFLP) analysis, PCR amplification of 16S rRNA genes from DNA was carried out with the primers FAM 63F (5′-CAGGCCTAACACATGGCAAGTC-3′) (Marchesi et al., 1998) and 518R (5′-CGTATTACCGCGGCTGCTCG-3′) (Lane, 1991). 50 μl reactions contained 0.4 μM forward and reverse primer, 0.1 mM dNTPs, 2.5U *Taq* polymerase, 5 μl of the reaction buffer supplied with the enzyme (Qiagen, Crawley, UK), and 1 μl of 10^–1^ dilution of DNA template. PCR amplification was carried out in an ABI 2720 Thermo-cycler (Applied Biosystems, Warrington, UK) as follows; 95°C for 2 min then 30 cycles of 95°C for 45 sec, 55°C for 1 min and 72°C for 30 s and a final extension step of 72°C for 10 min.

Amplified 16S rRNA genes were purified by using a Qiagen PCR purification kit (Qiagen, Crawley, UK) according to the manufacturer’s protocol and subsequently independently digested with AluI and CfoI (Roche Diagnostics, Basel, Switzerland) at 37°C for 3 h. 5 μl of each digest was desalted; glycogen (20 mg ml^–1^) (Thermo Scientific, Waltham, MA, USA) was added to a final concentration of 0.1 μg/ml and ethanol precipitated with 1/10 volume of 3M sodium acetate (pH 5.2) and 2 volumes of ice-cold ethanol. 0.5 μl of the desalted digestion reaction was added to 9.5 μl of deionised formamide and 0.5 μl ROX- labeled Genescan 500 internal size standard (both Applied Biosystems, Warrington, UK). Samples were denatured at 94°C for 5 min, cooled on ice and separated on an ABI 3700 (Applied Biosystems, Warrington, UK) using a 10 s injection and a 8.5 kV separation voltage.

Quantitative PCR was used to quantify 16S rRNA genes in sediments using an ABI Prism 7000 detection system as described by Smith et al. (2006) using the primers 1369F and 1492R and the *Taq*Man probe TM1389F (Table 1).

**TABLE 1:** **Q- (RT)-PCR primer and probe sets used in this study**.

**Target (reference)**	**Primer/probe^a^**	**Sequence (5′–3′)**	**Amplicon (bp)**	**Annealing°C**
16S rRNA	1369F	CGGTGAATACGTTCYCGG	123	56
Suzuki et al. (2000)	1492R	TACGGYTACCTTGTTACGACTT		
	TM1389F	CTTGTACACACCGCCCGTA		
*napA*-1	*napA-1F*	GTYATGGARGAAAAATTCAA	111	55
Smith et al. (2007)	*napA-1R*	GARCCGAACATGCCRAC		
	napA-1 TM*	AACATGACCTGGAAG		
*napA*-2	*napA-2F*	GAACCKAYGGGYTGTTATG	76	55
Smith et al. (2007)	*napA-2R*	TGCATYTCSGCCATRTT		
	*napA*-2 TM*	CTTTGGGGTTCAA		
*napA*-3	*napA*-3F	CCCAATGCTCGCCACTG	130	60
Smith et al. (2007)	*napA*-3R	CATGTTKGAGCCCCACAG		
	*napA*-3 TM*	TGGGTTGTTACGA		
*narG*-1	*narG*-1F	GACTTCCGCATGTCRAC	69	60
Smith et al. (2007)	*narG*-1R	TTYTCGTACCAGGTGGC		
	*narG*-1 TM*	TAYTCCGACATCGT		
*narG*-2	*narG*-2F	CTCGAYCTGGTGGTYGA	89	55
Smith et al. (2007)	*narG*-2R	TTYTCGTACCAGGTSGC		
	*narG*-2 TM*	AACTTCCGCATGGA		
*nrfA*-2	*nrfA*-2F	CACGACAGCAAGACTGCCG	67	60
Smith et al. (2007)	*nrfA*-2R	CCGGCACTTTCGAGCCC		
	*nrfA*-2TM*	TTGACCGTCGGCA		
*nirS*-e	*nirS*-ef-F	CACCCGGAGTTCATCGTC	172	60
Smith et al. (2007)	*nirS*-efR	ACCTTGTTGGACTGGTGGG		
	*nirS*-ef TM*	TGCTGGTCAACTA		
*nirS*-m	*nirS*-m-F	GGAAACCTGTTCGTCAAGAC	162	60
Smith et al. (2007)	*nirS*-mR	CSGARTCCTTGGCGACGT		
	*nirS*-m TM	TCTGGGCCGACGCGCCGATGAAC		
*nirS*-n	*nirS*-n-F	AAGGAAGTCTGGATYTC	140	55
Smith et al. (2007)	*nirS*-nR	CGTTGAACTTRCCGGT		
	*nirS*-n TM*	ATCCGAAGATSA		

*TM Taq*Man Minor Groove Binding; TM,* Taq*Man.*

### Q-(RT)-PCR of Nitrate and Nitrite Reducates Genes and Transcripts

Nitrate reductase genes (*narG* and *napA*) and nitrite reductase (*nirS* and *nrfA*) genes and transcripts were quantified from triplicate sediment samples from each site using Q-(RT)-PCR *Taq*Man assays as described in Smith et al. (2007, 2006). Briefly, *Taq*Man primer and probe sets targeting two *narG* (*narG*-1 and 2), three *napA* (*napA*-1, 2, and 3), three *nirS* (*nirS*-ef, *nirS*-m, *nirS*-n) and a single *nrfA* phylotype were targeted as genetic markers of nitrate reduction, denitrification and DNRA, respectively. *nirK* was targeted but it was not detected along the estuary at any of the time points in question (data not shown). For transcript quantification, *narG*-1 and *nirS*-ef gene transcripts were targeted at the EH and ME sites only based on results of our earlier study (Dong et al., 2009). Details of primer and probe sequences are provided in Table 1. Gene and transcript abundances were calculated from standard curves (Table 2).

**TABLE 2:** **Quantitative and reverse transcriptase quantitative-PCR standard curve descriptors**.

**Phylotype**	**Template**	***r*^2^**	**y-intercept**	***E* (%)**	**Ct-cutoff**
16S rRNA	DNA	0.998	35.1	92.6	28.9
*narG*-1	DNA	0.998	41.1	77.6	29.9
*narG*-2	DNA	0.998	43.4	85.4	ND
*napA*-1	DNA	0.994	44.7	95.5	32.3
*napA*-2	DNA	0.999	42.2	86.9	31.3
*napA*-3	DNA	0.997	41.9	89.9	34.1
*nirS*-ef	DNA	0.998	35.0	86.0	35.9
*nirS*-m	DNA	0.996	41.1	86.0	ND
*nirS*-n	DNA	0.998	42.8	82.1	ND
*nrfA*-2	DNA	0.997	39.9	96.0	28.5
*narG*-2	cDNA	0.999	43.3	83.0	ND
*nirS*-ef	cDNA	0.998	43.0	85.0	ND

E, amplification efficiency; ND, no template control not detected.

### Statistical Analyses

T-RFLP profiles were aligned on the basis of T-RF size in base pairs and the individual peak areas of the T-RFs identified by using T-Align (Smith et al., 2005) based on a 0.5-bp moving average, resulting in the generation of datasets of aligned T-RFs that gave individual relative peak areas as a proportion (%) of the overall profile. All T-RFs that contributed less than 1% of the total peak area for a profile were excluded from further analysis. The aligned T-RFs were transformed by log(X+1) to remove any weighting from dominant peaks and analyzed with a Bray-Curtis similarity matrix (Clarke et al., 2006) in Primer v6 (Primer-E, Plymouth, UK). The resultant similarity matrix was analyzed in a two-dimensional multidimensional scaling (MDS) plot.

Variation in nitrate reduction rates or gene abundances between sites and within sites at different months were analyzed using a one-way ANOVA followed by a *post hoc* test (Tukey, 1953) in SPSS v14. Data was log(x+1) transformed, as necessary. Spearman’s rank correlation analysis was performed to investigate correlations between denitrification or DNRA rates and gene abundances of corresponding genetic determinants in SPSS. A Bray-Curtis resemblance matrix (Clarke et al., 2006) of quantitative PCR gene abundances was generated from the log (x+1) transformed data and an Euclidean distance resemblance matrix was constructed from rate process data and nutrient concentrations and used to construct MDS. Variation among sites was assessed using ANOSIM a one-way analysis of similarity in Primer-6 (Clarke, 1993). BIO-ENV and LINKTREE were used to link gene abundance patterns with rate process and nutrient data. Seasonal trajectories were added to MDS plots by ordering sampling months numerically using the overlay trajectory function within PRIMER-6. MDS, ANOSIM, BIO-ENV, and LINKTREE analysis were carried out in Primer 6 (PRIMER-E Ltd, Plymouth Marine Laboratory, UK).

## Results

### *In situ* Nitrate Concentrations and Water Temperature

**FIGURE 1:**
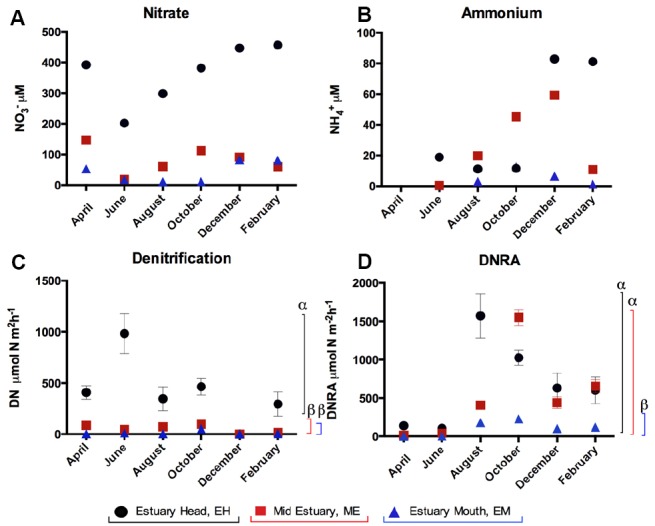
**Temporal and spatial variation in (A) nitrate and (B) ammonium concentrations and rates (±SE, *n* = 5) of (C) denitrification and (D) DNRA in sediments along the Colne estuary sampled from April 2005 to February 2006.** For each process, significant differences in the overall process rates between sites along the estuary are indicated by different Greek letters above the colored lines [black, estuary head (EH); red, mid-estuary (ME); and blue, estuary mouth (EM)].

Nitrate concentrations in water decreased from the EH to EM with an annual mean (± SE) of 399.7 ± 50.0 μM at EH, 98.8 ± 23.4 μM at ME, and 43.1 ± 10.2 μM at EM. (NO_3_^–^ and NH_4_^+^ concentrations for individual months are shown in Figures 1A,B). Water temperatures varied seasonally ranging from 4 to 19°C (in February and August respectively).

### Spatial and Temporal Variation in Rates of Benthic Denitrification and DNRA

Rates of benthic denitrification decreased from the head toward the mouth of the estuary (Figures 1C,D) following the nitrate concentration gradient in the water column. At the EH, the mean (±SE) annual rate of denitrification (415.6 ± 131.2 μmol N m^–2^ h^–1^) was significantly higher (ANOVA, *P* < 0.05) than ME (53.1 ± 16.1 μmol N m^–2^ h^–1^) and the EM (12.3 ± 8.2 μmol N m^–2^ h^–1^), while the latter two did not differ significantly from each other (Figure 1; *P* = 0.388). Mean annual rates of DNRA at the EH (679.1 ± 226.9 μmol N m^–2^ h^–1^) and ME (516.5 ± 230.1 μmol N m^–2^ h^–1^) did not differ significantly from each other (*P* > 0.05) but were significantly higher (Figure 1, *P* < 0.05) than at EM (106.3 ± 37.2 μmol N m^–2^ h^–1^). Rates of denitrification and DNRA at all three sites showed significant seasonal variability (*P* < 0.05, Figure 1). At each site, benthic rates of denitrification were greater than DNRA in April and June only. Rates of DNRA were greater than denitrification at all sites in August, October, December and February (Figure 1).

### Spatial and Temporal Variation in 16S rRNA Community Structure, Abundance, and Diversity

**FIGURE 2:**
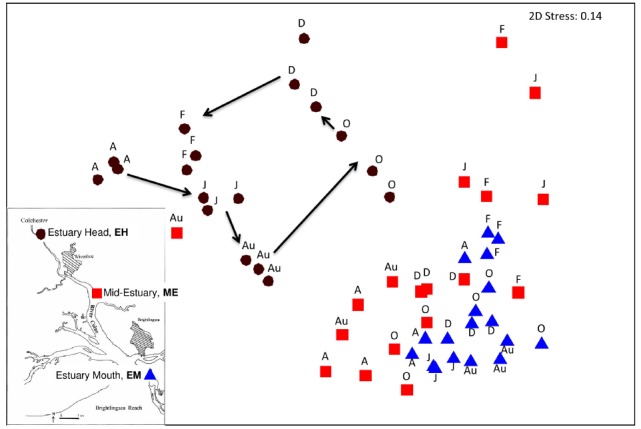
**Non-metric multi-dimensional scaling plot of total community T-RFLP profiles from three sites along the estuary sampled at 2-monthly intervals from April 2005 to February 2006.** Each month is represented in biological triplicates and coded according to site. Insert map showing sample location along the Colne estuary. Black circles represent the estuary head (EH), red squares mid-estuary (ME) and blue triangles the estuary mouth (EM). A, April; J, June; Au, August; O, October; D, December; F, February 2006.

Changes in community structure along the estuary over the year were assessed by 16S rRNA gene T-RFLP analysis. The results of a Bray-Curtis similarity matrix of T-RFLP profiles generated in triplicate from each site along the estuary over the year revealed two distinct clusters (Figure 2), with the EH forming a separate cluster from the lower estuary sites of ME and EM which were more similar to each other than to the EH (Figure 2). This separation were supported by ANOSIM analysis: EH verses ME *R* = 0.58, *P* < 0.001, EH verses EM *R* = 0.913, *p* < 0.001 and ME verses EM R = 0.218, *p* < 0.001. At the EH a sequential seasonal shift in community structure was observed as illustrated by the trajectory (Figure 2), but seasonal cycles in community structure were not evident at the other two sites.

**FIGURE 3:**
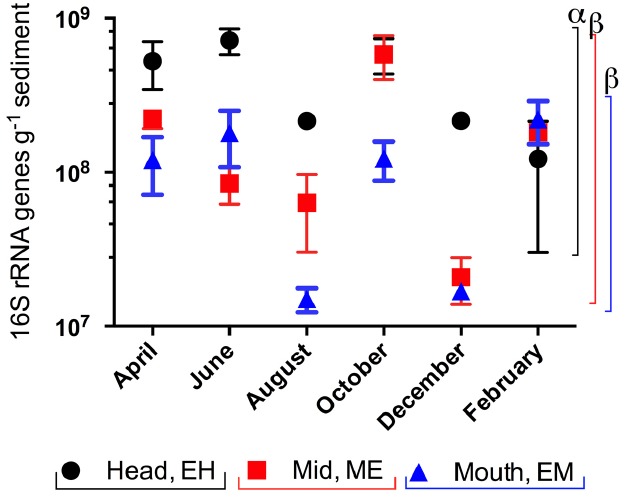
**Spatial and temporal variation in 16S rRNA gene copy abundances in sediments from the Colne estuary from April 2005 to February 2006.** Standard errors (*n* = 3) are shown. Greek letters above colored lines [black, estuary head (EH); red, mid-estuary (ME); and blue, estuary mouth (ME)] indicate statistical differences in the overall gene abundances across the year between sites (*p* < 0.001).

16S rRNA gene abundances at each site from April 2005 to February 2006 (Figure 3) indicated a significant site and time effect (two way ANOVA, *P* < 0.001). Gene abundances were highest at the EH and significantly higher than in sediments from ME and the EM (*P* < 0.001); while there were no significant differences between ME and EM sediments (*P* = 0.071). Within individual sites, there was only significant variation in 16S rRNA gene copy abundances between months at ME in October (ANOVA *P* < 0.008, Bonferroni correction).

### Spatial and Temporal Variation in the Abundance of Nitrate Reductase (narG and napA) and Nitrite Reductase (nirS and nrfA) Genes

**FIGURE 4:**
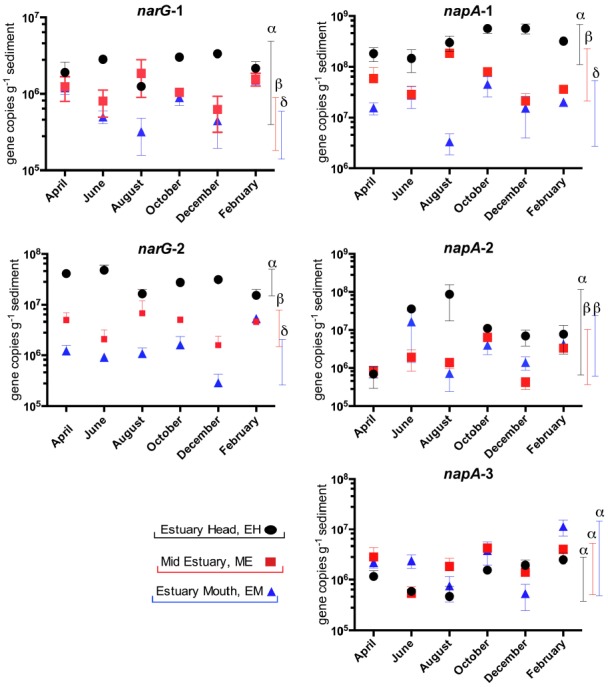
**Variation in abundance (gene copies g^–1^ sediment ±SE, *n* = 3) of nitrate reductase genes (*narG* and *napA*) in sediments along the Colne estuary sampled from April 2005 to February 2006.** For each phylotype, significant differences in annual gene abundances between sites along the estuary are indicated by different Greek letters above colored lines [black, estuary head (EH); red, mid-estuary (ME); and blue, estuary mouth (EM)].

**FIGURE 5:**
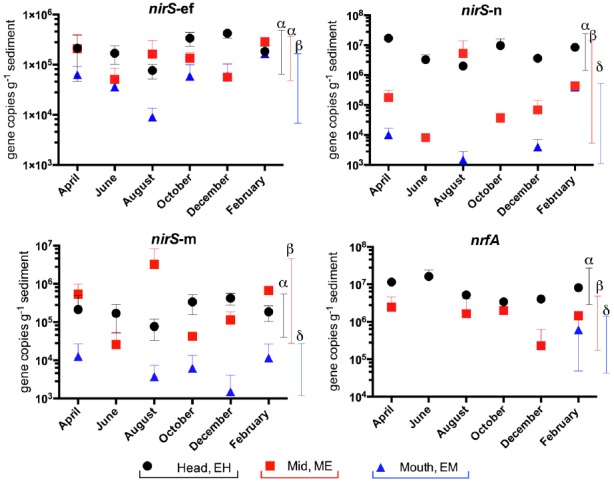
**Variation in abundance (gene copies g^–1^ sediment ±SE, *n* = 3) of nitrite reductase genes (*nirS* and *nrfA*) in sediments along the Colne estuary sampled from April 2005 to February 2006.** For each phylotype, significant differences in annual gene abundances between sites along the estuary are indicated by different Greek letters above colored lines [black, estuary head (EH); red, mid-estuary (ME); and blue, estuary mouth (EM)].

In our previous study of the Colne estuary, a suite of nine *Taq*Man primer and probe sets were designed targeting indigenous nitrate and nitrite reducing phylotypes present (Smith et al., 2007). These included two *narG* (*narG*-1 and 2), three *napA* (*napA*-1, 2, and 3), three *nirS* (nirS-e, m and n) and one *nrfA* (nrfA-2) gene targets. Nitrate (Figure 4) and nitrite (Figure 5) reductase gene abundances were greatest at the EH and lowest at the EM (*P* < 0.05) for eight of the nine phylotypes. The *napA*-3 phylotype was the exception, with no significant difference in gene abundances observed along the estuary (*P* < 0.05). Within individual sites there was only limited temporal variability in gene abundances for both nitrate and nitrite reductase phylotypes (Figures 4 and 5).

**FIGURE 6:**
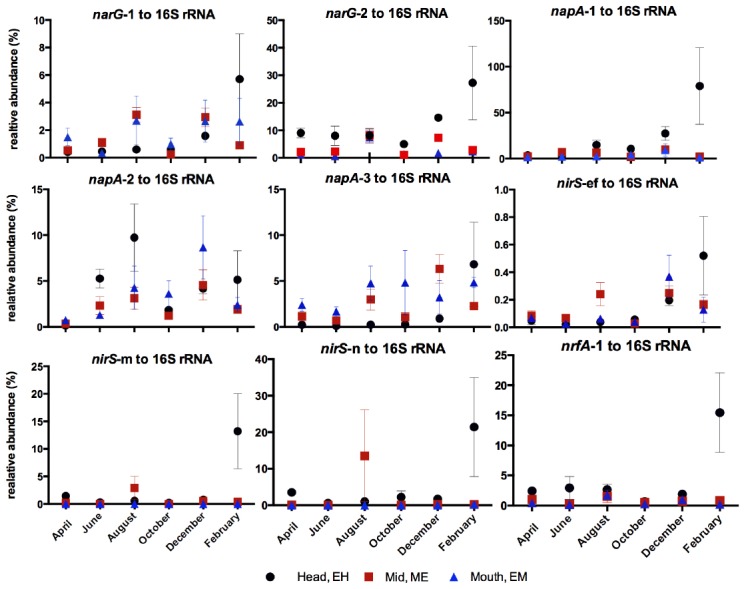
**Relative abundance (%) of nitrate- and nitrite- reductase phylotype gene abundances to 16S rRNA gene abundances along the estuary over the annual sampling period**.

Percentage relative abundance of nitrate- and nitrite- reducing functional gene abundances to 16S rRNA gene abundances were calculated for each phylotype (Figure 6). In general the highest relative abundance of nitrate- or nitrite-reducing functional gene abundances to 16S rRNA gene abundances were observed at the EH (Figure 6) indicating this site as having not only the most abundant bacterial community but also the highest proportion of nitrate reducers in that community: commensurate with the highest nitrate concentrations along the estuary. Exceptions to this trend were the *narG*-1 and *napA*-3 phylotypes, which were greatest at ME and EM sites respectively. For all genes a peak in relative abundance was observed at the EH in February 2006 corresponding to a peak in nitrate and ammonium concentrations (Figure 1).

### Spatial and Temporal Variation in narG and nirS Gene Transcription

**FIGURE 7:**
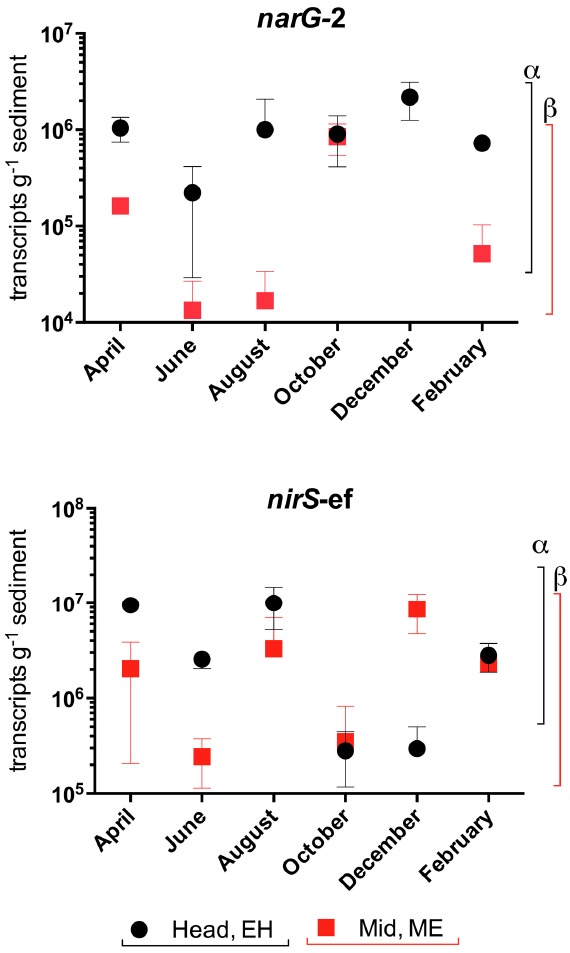
**Variation in nitrate (*narG*-2) and nitrite (*nirS*-ef) reductase gene transcript abundances (transcripts g^–1^ sediment +/–SE, *n* = 3) in sediments along the Colne estuary sampled from April 2005 to February 2006.** For each phylotype, significant differences in annual transcript abundances between sites along the estuary are indicated by different Greek letters above the colored lines representing each site [black, estuary head (EH); red, mid-estuary (ME)].

Trends of transcript abundances reflected those observed at the DNA level—*narG* transcript abundances were highest at the EH, while differences in *nirS*-ef transcripts numbers were not observed between sites (Figure 7, *P* > 0.05).

### Inter-relationships between Functional Gene Abundances, Nitrate Concentrations, Rates of Denitrification and DNRA

**FIGURE 8:**
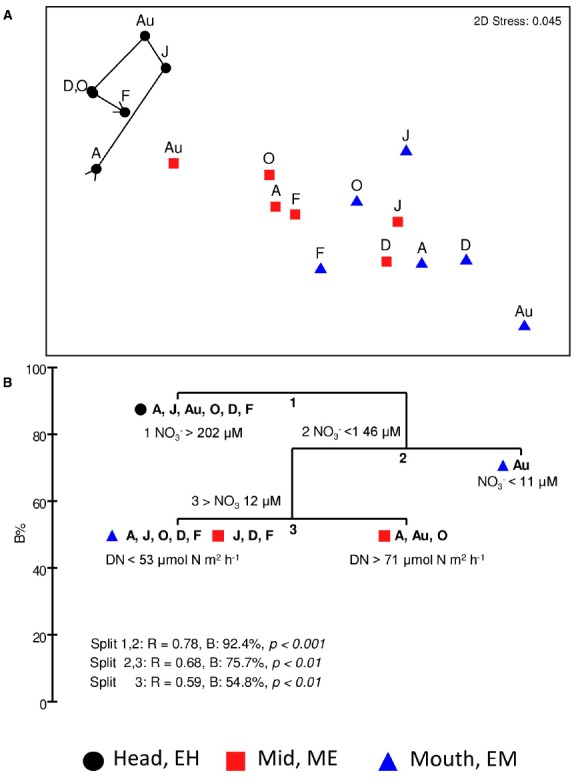
**(A)** Non-metric multidimensional scaling (MDS) ordination of a Bray-Curtis resemblance matrix calculated from square-root transformed mean Q-PCR nitrate- and nitrite-reductase gene abundances quantified at 2-monthly intervals from sediments at each site; temporal variation at the estuary head (EH) is illustrated by a time-line trajectory drawn through sampling time points. **(B)** LINKTREE analysis of all sites and time points to identify environmental variable range (nitrate, ammonia, denitrification or DNRA) driving clustering of nitrate-nitrite reducing community observed in 8A. Three splits (1–3) and variable range responsible for the divide are shown. %B, an absolute measure of group difference is shown on the x-axis. ANOSIM R value and *p*-values are reported for each split. Months: A, April; J, June; Au, August; O, October; D, December; F, February (2006).

An MDS plot of Bray-Curtis square-root transformed mean gene abundances (i.e., four genes; totalling nine phylotypes) at each site from April to February (Figure 8A) shows spatial and temporal variation in the abundance of the nitrate- and nitrite-reductase genes along the estuary. Gene abundances at the EH site clustered separately from those at ME and EM sites. A one-way ANOSIM indicated a significant difference in the abundance of nitrate- and nitrite-reductases genes at the three sites (*R* = 0.679, *P* < 0.001). As with the 16S rRNA community analysis (Figure 2), there was evidence of community change over time (seasonality) at the EH in nitrate- and nitrite reductase gene abundances as indicated by the trajectory on Figure 8A. Relationships between gene abundances, denitrification and DNRA rates and nitrate and ammonia concentrations were explored using BIO-ENV and LINKTREE in PRIMER-6 (Clarke and Ainsworth, 1993). BIO-ENV identified nitrate (*R* = 0.639, *p* < 0.001) as the single measured abiotic variable that best explained the clustering of nitrate and nitrite reductase gene abundances along the estuary. To further explore the observed clustering of nitrate and nitrite reductase gene abundances, a LINKTREE non-parametric analysis was conducted in PRIMER6 (Figure 8B), to link the gene abundance clusters to the range of nutrient concentrations or rates of denitrification and DNRA. Three distinct splits labeled 1, 2, and 3, formed in the LINKTREE dendrogram. The first split was observed between the EH and the lower estuary sites and was defined by NO_3_^–^ concentrations > 202 μM. All other sites, fell into split two where NO_3_^–^ concentrations were < 146 μM. The next split separated the EM site in August from the rest based on the lowest observed nitrate concentration. Split 3, divided EM sites in April, August and October based on denitrification rates > 71 μmol N m^2^ h^–1^ with remaining sites characterized by rates of denitrification below < 53 μmol N m^2^ h^–1^.

Spearman’s correlation co-efficient analysis was preformed to examine correlations between rates of denitrification or DNRA and the abundance of their corresponding genetic determinants (Table 3). For denitrification significant weak to strong (correlations absolute value of r range between 0.40 and 0.79) were observed between the rates of denitrification and corresponding abundances of *nirS*-n and *nirS-m* phylotypes. *nirS-n*, *m*, and *e* primer and probe sets target *nirS* phylotypes first recovered as mRNA *nirS* gene sequences from sediments at the head of the Colne estuary similar to *nirS* from gamma and alpha proteobacteria (Nogales et al., 2002; Smith et al., 2007). Weak to moderate, but not significant correlations were observed between denitrification and *nirS-e* and DNRA and *nrfA* gene abundances (Table 3). The *nirS*-e primer and probe set target *nirS* gene sequences first retrieved from the ME site of the Colne as mRNA and phylogenetically group with gamma proteobacteria (Nogales et al., 2002; Smith et al., 2007). The *nrfA* primer set targeted *nrfA* phylotypes retrieved from the Colne EH site that phylogenetically group with epsilon-proteobacteria (Smith et al., 2007).

**TABLE 3:** **Spearman’s rank correlation co-efficient between rate process measurements and functional gene abundances**.

**Rate Process**	**gene**	***r***	***P***	***n***
Denitrification	*nirS-*ef	0.422	0.081	18
	*nirS-n*	0.562	0.015*	18
	*nirS-m*	0.608	0.007*	18
DNRA	*nrfA*	0.485	0.056	18

*P < 0.05.

## Discussion

In this study, we report spatial and temporal dynamics in the activity of benthic nitrate reduction coupled to quantification of nitrate reducing functional genes and transcripts along an estuarine gradient. Denitrification, DNRA and corresponding nitrate and nitrite functional gene abundances decreased along the estuary, following the nitrate gradient in the water column as previously observed in the Colne estuary (Ogilvie et al., 1997; Dong et al., 2009) and the Thames (Trimmer et al., 2000). Denitrification at the EH greatly exceeded that of the other sites. At the EH, rates of denitrification were highest in June, whereas in the middle of the estuary and at the mouth rates peaked in October. Lowest rates of denitrification at all three sites were observed in December. Our earlier studies of the Colne (Dong et al., 2000) and the Great Ouse (Trimmer et al., 1998) had shown highest denitrification rates during late spring/summer and lowest denitrification activity during the winter.

The separation of the EH from sites lower down the estuary was not as pronounced when it came to DNRA. Rates of DNRA at the EH and middle sites were similar, and DNRA at the middle estuary site was, in fact, greater than at the EH in four out of six months (October and February). Seasonal variability was also observed for DNRA, with the highest rates in late summer and early autumn, and lowest rates in spring (Figure 1). Giblin et al. (2010) measured denitrification and DNRA in the Parker River estuary for 13 years at a single upstream site (salinity between 0 and 18 ppt) between 1993 and 2006. Large seasonal and inter-annual variation in denitrification and DNRA was reported, primarily driven by salinity not temperature. Rates of DNRA in the Parker River Estuary were highest in late summer (August) and lowest in early spring (March). Spatially, denitrification in the Parker River exhibited the opposite trend to the Colne estuary, as it was inversely related to the salinity gradient with a peak in denitrification in late spring in this estuary. In the Norsminde Fjord, Denmark, Jørgensen (1989) reported two maxima in denitrification, the first in early spring and the second in autumn. A peak in DNRA was observed in late summer, attributed to the more highly reduced sediments within the estuary at this time. The results from the Colne data are in agreement with modeling studies (Kelly-Gerreyn et al., 2001), and data from tropical estuaries (Dong et al., 2011) that suggested DNRA tends to become increasingly important at higher environmental temperatures.

Denitrification has in the past been considered the dominant nitrate reduction pathway in coastal and marine sediments and DNRA less important, if considered at all (for a review, see Burgin and Hamilton, 2007; Giblin et al., 2013). Our previous single time-point study of denitrification, DNRA and anammox, showed that more nitrate was removed from the system via denitrification than was reduced to ammonia by DNRA (Dong et al., 2009). Anammox was detected only at the EH and accounted for 30% of the N_2_ formation. In this study anammox was not measured, due to logistical limitations, instead focusing on nitrate reduction pathways of denitrification and DNRA. Over the annual time period, denitrification rates exceeded those of DNRA only in the late spring/early summer (April and June) at all three sites along the estuary. For the remainder of the year, at the time points measured, rates of DNRA exceeded those of denitrification indicating that more nitrate was being reduced and converted to ammonia than was being lost from the system in gaseous forms via denitrification. This further highlights the importance of not relying on single time point studies to understand the dynamics of the nitrogen cycle in dynamic estuarine systems.

Denitrification and DNRA compete for nitrate and carbon within sediments. The availability of nitrate and organic carbon are key factors controlling rates of benthic denitrification (Cornwell et al., 1999; Dong et al., 2000; Fulweiler and Heiss, 2014). Previous studies of the Colne estuary have indicated that denitrification at the EH is carbon limited, while denitrification ME and at the EM is nitrate limited (Papaspyrou et al., 2014). However, the ratio of electron donor to acceptor can influence the pathway and fate of nitrate. DNRA has a higher affinity for nitrate than denitrification and may be favored in nitrate-limited, carbon-rich environments (King and Nedwell, 1985; Burgin and Hamilton, 2007; Kraft et al., 2014). This is due to the requirement of only 5 electrons to reduce nitrate in denitrification verses the eight required for DNRA (Tiedje, 1988). DNRA may therefore outcompete denitrification in nitrate-limited environments where these organisms gain more energy from DNRA than denitrifiers can from denitrification. Indeed nitrate concentrations at the ME site were much lower than at the EH and in the months where DNRA was the dominant process it was 5–2120 times greater than denitrification at this site (Alresford). Recent studies in similar environments have shown that DNRA is often the prominent nitrate reduction pathway. For examples, Giblin et al., 2013, showed DNRA was greater than denitrification in 30% of 55 coastal sediments sites examined. Similarly, Song et al. (2014), in a single time point study of benthic DNRA in the New River estuary, North Carolina, USA showed it was responsible for 44–74% of nitrate reduction and reported that DNRA rates were greater than denitrification (Lisa et al., 2014).

The results of our study have highlighted, for the first time, the importance of DNRA as a significant pathway for benthic sediment nitrate reduction in the Colne estuary. The rates of benthic DNRA of nitrate from the water column (DNRA_w_) in the whole estuary was estimated as 11.48 Mmol N year^–1^ derived by multiplying the mean annual rates of DNRA at the EH (679 μmol N m^–2^ h^–1^), ME (517 μmol N m^–2^ h^–1^), and EM (106.7 μmol N m^–2^ h^–1^) by the total area of sediment (which is defined as the 72% of the area totally immersed at spring tide; Ogilvie et al., 1997) in the sector of the river center around each site (Dong et al., 2000). Using the mass balance approach, by subtracting oxidized inorganic nitrogen (NO_3_^–^ + NO_2_^–^) removal by denitrification supported by nitrate from water column (5.09 Mmol N year^–1^) from the total sediment uptake of oxidized inorganic nitrogen (16.28 Mmol N year^–1^), a very similar DNRA rate of 11.19 Mmol N year^–1^ was obtained (Thornton et al., 2007).

At the molecular level, the abundances of gene and transcript molecular markers for nitrate reduction (*nar*G and *nap*A), denitrification (*nirS*) and DNRA (*nrf*A) generally showed a consistent overall spatial trend of declining abundances from the EH to the EM (Figures 4–6) as observed in the rate process data, supporting and extending our previous studies along the Colne estuary at single time points (Smith et al., 2007; Dong et al., 2009). In contrast to our observations in the hypernutrified Colne, *nirS* and *nirK* gene abundances along the lower nutrient Fitzroy estuary, Australia showed no statistical difference between sites, despite some observed variability in the net rates of denitrification along the estuary (Abell et al., 2010). The one exception to this trend of decreasing gene abundances along the estuary was the *napA*-3 phylotype (alphaproteobacteria) where gene abundances throughout the 12 month sampling period remained constant along the estuary gradient (Figure 4), suggesting a different selective mechanism and different ecological significance for this *nap* phylotype than for the other two phylotypes (both gammaproteobacteria). While we have determined the distributions of these different functional genes and their phylotypes along the estuary, one of the key questions remaining to be elucidated is the different functions of these phylotypes, which can probably only be clarified by controlled laboratory-based studies. Richardson et al. (2001) suggested that NAR and NAP provide adaptations to different nitrate environments, the former facilitating nitrate reduction in reduced, high nitrate environments whereas NAP is adaptive to effective nitrate scavenging in lower nitrate and less reduced environments. The distributions that we measured along the Colne estuary would suggest that these different *napA* phylotypes, particularly *napA-3* generally support this hypothesis as total bacterial numbers decline along the estuary but *napA* numbers remain high indicating that they are relatively more important at lower nitrate concentrations than *narG*.

While gene and transcript abundances generally decreased along the estuary gradients, as observed in the rate process data, finer scale correlations between rates and gene/transcript abundances on temporal scales were not observed. Some studies have shown good agreement between activity measurements and gene and/or transcript abundances, e.g., in studies of archaeal nitrification (Wuchter et al., 2006). However, there is evidence in the literature to indicate the direct measurement of functional genes at DNA and even mRNA levels can be uncoupled to activity measurements, indicating that substantial post-transcriptional, protein assembly and/or environmental factors ultimately control activity. For example, Ikeda et al. (2009) examined the roles of NAR and NAP in nitrate reduction in *Pseudomonas sp*. and found that nitrate reductase activity and *napA* or *narG* gene transcription were not necessarily positively correlated, leading them to conclude that there were subsequent post-translational modifications even in pure culture. In soils, Liu et al. (2010) demonstrated the dramatic effect increasing pH had on reducing denitrification activity, yet this trend was not reciprocated in *nirS* and *nosZ* gene and transcript abundance, leading them to conclude that reduction in pH affected denitrification after transcription. In the highly nutrified Colne estuary, the high nitrate concentrations in the water are drastically reduced within the surface sediment by rapid nitrate reduction in the sub-oxic zone (the surface oxic layer of sediment is usually <2–3 mm depth (Robinson et al., 1998). Consequently, benthic nitrate reducers are operating at very low pore water nitrate concentrations where rates of nitrate reduction approximate to first order kinetics; well below any nitrate-saturating concentrations when nitrate reduction might correlate with genetic potentials. The correspondence between rate processes and gene abundances/transcripts is likely to be closer the nearer *in situ* substrate concentrations are to saturating concentrations of substrates. Furthermore other physical-chemical factors such as temperature or pH may control key enzyme activity, and *in situ* rates, without necessarily directly affecting transcription. Further and future studies will focus on determining the links between environmental conditions, nitrate and nitrite reducing communities and rates of nitrate reduction and controlling factors influencing the fate of nitrate within estuarine sediments.

### Conflict of Interest Statement

The authors declare that the research was conducted in the absence of any commercial or financial relationships that could be construed as a potential conflict of interest.
